# Utility values for age-related macular degeneration patients in Korea

**DOI:** 10.1371/journal.pone.0201399

**Published:** 2018-07-31

**Authors:** Seulggie Choi, Sang Min Park, Donghyun Jee

**Affiliations:** 1 Seoul National University Graduate School, Department of Biomedical Sciences, Seoul, Republic of Korea; 2 Seoul National University Hospital, Department of Family Medicine, Seoul, Republic of Korea; 3 St. Vincent’s Hospital, College of Medicine, Division of Vitreous and Retina, Department of Ophthalmology, Catholic University of Korea, Suwon, Republic of Korea; Universita degli Studi di Firenze, ITALY

## Abstract

**Purpose:**

Age-related macular degeneration (AMD) is one of the most important causes of blindness globally and may lead to decreased quality of life. Utility values for AMD patients according to sociodemographic and clinical characteristics have been little-studied, particularly among Asian populations.

**Methods:**

A total of 1,283 AMD patients were identified from the Korean National Health and Nutrition Examination Survey from 2008 to 2012. A 45-degree digital retinal image for each eye was used to identify AMD patients. The utility values, calculated by the three level version of EuroQol-5D, of AMD patients according to sociodemographic and clinical characteristics were determined. The Kruskal-Wallis test was used to identify factors associated with reduced utility values among AMD patients.

**Results:**

The mean utility value for AMD patients was 0.8765. Patients who were older (mean utility value 0.8339), were women (0.8488), had lower education levels (0.8287), were not employed (0.8467), and had lower household income (0.8022) had lower utility values (all p values <0.001). Utility values did not significantly differ according to AMD subtype (p value 0.729), likely due to the lack of enough power as only 48 patients had late AMD. Patients with lower best-eye visual acuity (BEVA) had lower utility values compared to those with high BEVA, even among those with high worst-eye visual acuity (WEVA) (p value <0.001).

**Conclusion:**

Sociodemographic factors and visual acuity are important factors in determining the quality of life among AMD patients. Preserving BEVA, regardless of WEVA, may be associated with improved quality of life.

## Introduction

Age-related macular degeneration (AMD) is the most common cause for blindness in developed countries [1]. A progressive disorder characterized by atrophy of the macula and central vision function impairment, AMD primarily affects the elderly population [2]. Due to the ever-increasing proportion of elderly adults, the incidence and prevalence of AMD are expected to increase globally [3]. Due to the fact that AMD may lead to serious vision impairment, one of the primary concerns for AMD patients is reduced quality of life. Therefore, a major therapeutic goal for AMD management is maintaining the quality of life of the patients at a socially acceptable cost. Identifying and controlling factors associated with reduced utility values among AMD patients are needed in order to maximize the quality of life for AMD patients.

Although multiple previous studies have evaluated the utility values for AMD patients, most studies focused on visual acuity of AMD patients [4, 5]. However, sociodemographic factors such as household income and education are also important contributors to utility values, indicating the need for studies investigating the impact of sociodemographic factors on utility values among AMD patients. Moreover, there are limited studies on utility values for AMD patients within Asian populations. Multiple reports have shown that AMD patients within Asian populations differ from those within Western populations in prevalence of AMD subtype, which could lead to different utility values among Asian populations [6]. Furthermore, as the innate differences in sociocultural and healthcare across regions may lead to different utility values, studies evaluating the quality of life among AMD patients within Asian populations are needed.

Therefore, using a nationwide representative survey in Korea, we investigated the sociodemographic and clinical factors associated with utility values among AMD patients.

## Materials and methods

### Study population

The Korean National Health and Nutrition Examination Survey (KNHANES) database conducted from 2008 to 2012 was used for this study. A nationwide population-based cross-sectional survey consisting of health records from health interviews and examinations, KNHANES is conducted by the Division of Chronic Disease Surveillance from the Korea Centers for Disease Control and Prevention. KNHANES represents the entire non-institutionalized population in Korea since approximately 4,000 households in 200 enumeration districts are selected annually by a stratified, multistage, clustered sampling method. All participants signed informed consent forms agreeing to the use of their information in this survey. The Korean Ophthalmologic Society had participated in KNHANES from 2008 to 2012, which led to inclusion of ophthalmologic interviews and examinations conducted by trained ophthalmologists.

All participants underwent a thorough ophthalmologic examination, which included a 45-degree digital retinal image for each eye. Early AMD was defined as the presence of a soft drusen or any drusen with pigmentation changes in the macular retinal pigment epithelium (RPE). Wet AMD was defined when detachment of the RPE or sensory retina, or subretinal epithelial hemorrhages or scars were observed. Dry AMD was defined upon retinal depigmentation spanning ≥175 μm in diameter with visible choroidal vessels. A detailed description of defining AMD patients can be found in a previous study [7]. Finally, visual acuity was measured at 4 meters with an international standard vision chart based on the LogMAR Scale (Jin’s Vision Chart, Seoul, Korea).

Among the 26,622 participants aged 19 years or more with data available on fundus images, 25,314 participants who did not meet the criteria for having AMD were excluded, resulting in 1,308 AMD patients (prevalence rate 4.9%). Among them, 4 participants without values on education status, 20 participants without values on household income, and 1 participant without values on martial status were excluded, ultimately resulting in a study population of 1,283 AMD patients.

### Measurement of utility value

The three-level version of EuroQol-5D (EQ-5D-3L), developed by the EuroQol group, is a generic preference-based measure consisting of five questions that reflect the current health status of the patient. The questions are composed of five dimensions: mobility, self-care, usual activities, pain/discomfort, and anxiety/depression. Each question has three levels that indicate (a) no problem, (b) some problems, or (c) severe problems. A possible 243 health states can be described by EQ-5D-3L based on the five dimensions and three levels for each dimension. EQ-5D-3L have been widely used as a method of assessing the health status. EQ-5D-3L is useful for comparison across regions due to its brevity, making it possible to compare utility values across countries and populations. Following the EuroQol group-recommended procedure, the Korean EQ-5D-3L was developed by the Korean Centers for Disease Control and Prevention. The range for the Korean EQ-5D-3L utility score is from -0.171 (worst health status) to 1.000 (best health status). EQ-5D-3L is a continuous measure of utility, with values closer to 1.000 indicating the highest utility values. Since EQ-5D-3L is a continuous measure, no particular cut-off points exist that indicate a certain level of utility, but rather is useful in comparing utility values between certain groups by mean values.

### Demographics and measures of AMD severity

Among patient demographics, age (years, less than 50, 50–59, 60–69, and 70 or more), sex (men and women), education (elementary school or lower, middle school, high school, technical college, and college or higher), employment status (yes and no), household income (1^st^, 2^nd^, 3^rd^, and 4^th^ quartiles), and marital status (yes and no) were determined by a questionnaire. AMD subtype (early and late), and vision loss (no vision loss and vision loss) defined when visual acuity was measured at less than 0.1 in either eye, were determined by ophthalmologic examinations. Based on the visual acuity determined by ophthalmologic examinations, patients were categorized into 20/20 to 20/25, 20/30 to 20/40, 20/50 to 20/100, and ≤20/200 for both best-eye visual acuity (BEVA) and worst-eye visual acuity (WEVA). Finally, participants were divided into ≥20/40:≥20/200, ≥20/40:<20/200, <20/40≥20/200, and <20/40:<20/200 for the ratio between BEVA and WEVA values.

### Statistical analysis

The proportions of the patient demographics and measures of AMD severity were calculated in the number of patients and percent. For each patient demographic and measures of AMD severity, the mean, standard deviation (SD), and 95% confidence intervals (CI) of EQ-5D-3L utility values were calculated. Comparisons of utility values were done by the Kruskal-Wallis test due to the expected non-normal distribution of values. For the analysis of utility values according to sociodemographic factors, participants without AMD (n = 24,691) were separately used to determine the utility values according to sociodemographic factors. Statistical significance was determined at a *p* value of less than 0.05. All statistical analyses were conducted with STATA 13.0 (StataCrop LP, College Station, TX, USA).

### Ethical considerations

Informed consent was obtained for all participants of KNHANES from 2008 to 2012 before the survey. Since KNHANES is a publicly available database from the Korea Centers for Disease Control and Prevention, approval from the Institutional Review Board was not needed. All research methods adhered to the tenets of the Declaration of Helsinki.

## Results

Table 1 depicts the descriptive characteristics of the study population. The mean (SD) age for 1,238 AMD patients was 66.1 (10.2) years. There were more women (58.0%) than men (42.0%). The largest group of AMD patients had education levels of elementary school or lower (40.1%), while only 8.5% of the study population graduated from college or higher. There were slightly more unemployed AMD patients (52.8%) compared to those who were employed (47.2%). Most AMD patients were married (99.5%), had early AMD (91.4%), and did not have any vision loss (96.3%). The mean age for 24,691 non-AMD participants was 48.4 (16.0) years. Non-AMD participants were generally women (57.8%), employed (60.5%), and married (85.1%).

**Table 1:** Descriptive characteristics of the study population.

Descriptive characteristics	AMD patients (n = 1,283)	Non-AMD participants (n = 24,691)
Age, years, mean (SD)	66.1 (10.2)	48.4 (16.0)
Age, years, N (%)		
Less than 50	81 (6.3)	13,207 (53.5)
50–59	231 (18.0)	4,644 (18.8)
60–69	439 (34.2)	3,891 (15.8)
70 or more	532 (41.5)	2,949 (11.9)
Sex, N (%)		
Men	539 (42.0)	10,430 (42.2)
Women	744 (58.0)	14,261 (57.8)
Education, N (%)		
Elementary school or lower	515 (40.1)	3,811 (15.4)
Middle school	303 (23.6)	3,378 (13.7)
High school	257 (20.0)	5,609 (22.7)
Technical college	99 (7.7)	4,782 (19.4)
College or higher	109 (8.5)	7,111 (28.8)
Employment status, N (%)		
Yes	606 (47.2)	14,936 (60.5)
No	677 (52.8)	9,755 (39.5)
Household income, N (%)		
1^st^ quartile (lowest)	322 (25.1)	6,675 (27.0)
2^nd^ quartile	334 (26.0)	5,783 (23.4)
3^rd^ quartile	309 (24.1)	6,111 (24.8)
4^th^ quartile (highest)	318 (24.8)	6,122 (24.8)
Marital status, N (%)		
Yes	1,276 (99.5)	21,003 (85.1)
No	7 (0.6)	3,688 (14.9)
AMD subtype, N (%)		
Early AMD	1,173 (91.4)	-
Late AMD	110 (8.6)	-
Vision loss, N (%)		
No vision loss	1,235 (96.3)	-
Vision loss	48 (3.7)	-
BEVA		
20/20 to 20/25	798 (62.2)	-
20/30 to 20/40	326 (25.4)	-
20/50 to 20/100	147 (11.5)	-
≤20/200	12 (0.9)	-
WEVA		
20/20 to 20/25	582 (45.4)	-
20/30 to 20/40	335 (26.1)	-
20/50 to 20/100	270 (21.0)	-
≤20/200	98 (7.5)	-

Acronyms: SD, standard deviation; AMD, age-related macular degeneration

The utility values for AMD patients according to sociodemographic characteristics are shown in Table 2. The utility value for AMD patients decreased with increasing age (*p* value <0.001). Men and women had significantly different utility values (*p* value <0.001), with utility values of 0.9148 for men and 0.8488 for women. Higher education levels was significantly associated with greater utility values (*p* value <0.001), with those having graduated from college or higher having a mean EQ-5D-3L score of 0.9702. Employed and unemployed patients had mean EQ-5D-3L scores of 0.9099 and 0.8467, respectively (*p* value <0.001). Finally, higher household income was associated with greater utility values (*p* value <0.001), with patients within the highest quartile of household income having a mean EQ-5D-3L score of 0.9267. Similarly, non-AMD participants who were younger, male, had higher education, were employed, had higher household income, and were not married tended to have higher utility values (all *p* values <0.001).

**Table 2:** Utility values according to sociodemographic characteristics.

	EQ-5D-3L	
	Mean ± SD (95% CI)	
Category	AMD-patients	Non-AMD participants
All	0.8765±0.1734 (0.8671–0.8860)	0.9400±0.1182 (0.9385–0.9415)
Age		
Less than 50 years	0.9499±0.1266 (0.9219–0.9778)	0.9746±0.0629 (0.9735–0.9757)
50–59 years	0.9240±0.1437 (0.9054–0.9427)	0.9426±0.0998 (0.9397–0.9454)
60–69 years	0.8897±0.1591 (0.8747–0.9046)	0.9017±0.1357 (0.8974–0.9059)
70 years or more	0.8339±0.1913 (0.8176–0.8502)	0.8318±0.2007 (0.8246–0.8391)
*p* value	<0.001	<0.001
Sex		
Men	0.9148±0.1511 (0.9020–0.9276)	0.9571±0.1008 (0.9552–0.9590)
Women	0.8488±0.1831 (0.8357–0.8620)	0.9275±0.1281 (0.9254–0.9296)
*p* value	<0.001	<0.001
Education		
Elementary school	0.8287±0.2003 (0.8113–0.8460)	0.8507±0.1839 (0.8449–0.8566)
Middle school	0.8609±0.1713 (0.8415–0.8803)	0.8989±0.1429 (0.8941–0.9037)
High school	0.9257±0.1288 (0.9099–0.9415)	0.9558±0.0918 (0.9534–0.9582)
Technical college	0.9426±0.1030 (0.9221–0.9632)	0.9664±0.0769 (0.9642–0.9686)
College or higher	0.9702±0.0687 (0.9572–0.9833)	0.9772±0.0544 (0.9760–0.9785)
*p* value	<0.001	<0.001
Employment status		
Yes	0.9099±0.1327 (0.8893–0.9205)	0.9585±0.0860 (0.9572–0.9599)
No	0.8467±0.1984 (0.8317–0.8616)	0.9117±0.1507 (0.9087–0.9147)
*p* value	<0.001	<0.001
Household income		
1^st^ quartile (lowest)	0.8022±0.2208 (0.7780–0.8264)	0.8819±0.1697 (0.8779–0.8860)
2^nd^ quartile	0.8754±0.1603 (0.8582–0.8927)	0.9515±0.0938 (0.9490–0.9539)
3^rd^ quartile	0.9036±0.1510 (0.8867–0.9205)	0.9644±0.0782 (0.9624–0.9664)
4^th^ quartile (highest)	0.9267±0.1191 (0.9136–0.9399)	0.9682±0.0733 (0.9664–0.9701)
*p* value	<0.001	<0.001
Marital status		
Yes	0.8762±0.1737 (0.8667–0.8857)	0.9339±0.1245 (0.9322–0.9356)
No	0.9420±0.0854 (0.8630–1.0210)	0.9749±0.0626 (0.9728–0.9769)
*p* value	0.352	<0.001

*p* value calculated by Kruskal-Wallis test

Acronyms: EQ-5D-3L, three level version of EuroQol-5D; SD, standard deviation; CI, confidence interval

Table 3 shows the utility values for AMD patients according to AMD subtype and vision loss. Early and late AMD patients had mean EQ-5D-3L scores of 0.8767 and 0.8753, respectively. Patients without vision loss and vision loss had mean utility values of 0.8776 and 0.8484, respectively. There were no significant differences in EQ-5D-3L scores of patients according to AMD subtype (*p* value 0.729) and vision loss (*p* value 0.303). Utility values for AMD patients according to visual acuity are depicted in Table 4. For both BEVA and WEVA, lower visual acuity was associated with reduced EQ-5D-3L scores (both *p* values <0.001). Lower BEVA, regardless of WEVA, was associated with lower mean utility values (0.8082 for BEVA:WEVA <20/40:≥20/200 and 0.8093 for BEVA:WEVA <20/40:<20/200). Patients with lower BEVA had reduced utility values regardless of WEVA (*p* value <0.001).

**Table 3:** Utility values according to AMD subtype and vision loss.

Category	EQ-5D-3L
	Mean ± SD (95% CI)
AMD subtype	
Early AMD	0.8767±0.1721 (0.8668–0.8865)
Late AMD	0.8753±0.1874 (0.8399–0.9107)
*p* value (Kruskal-Wallis test)	0.729
Vision loss	
No vision loss	0.8776±0.1722 (0.8680–0.8873)
Vision loss	0.8484±0.2010 (0.7900–0.9067)
*p* value (Kruskal-Wallis test)	0.303

Acronyms: EQ-5D-3L, three level version of EuroQol-5D; SD, standard deviation; CI, confidence interval; AMD, age-related macular degeneration

**Table 4:** Utility values according to visual acuity.

Category	EQ-5D-3L
	Mean ± SD (95% CI)
BEVA	
20/20 to 20/25	0.9019±0.1460 (0.8918–0.9121)
20/30 to 20/40	0.8477±0.1872 (0.8273–0.8681)
20/50 to 20/100	0.8095±0.2305 (0.7719–0.8470)
≤20/200	0.7942±0.2833 (0.6141–0.9742)
*p* value (Kruskal-Wallis test)	<0.001
WEVA	
20/20 to 20/25	0.9148±0.1323 (0.9040–0.9255)
20/30 to 20/40	0.8584±0.1848 (0.8385–0.8782)
20/50 to 20/100	0.8274±0.2057 (0.8028–0.8521)
≤20/200	0.8464±0.2023 (0.8055–0.8874)
*p* value (Kruskal-Wallis test)	<0.001
BEVA:WEVA	
≥20/40:≥20/200	0.8870±0.1600 (0.8774–0.8965)
≥20/40:<20/200	0.8629±0.1860 (0.7990–0.9268)
<20/40:≥20/200	0.8082±0.2341 (0.7699–0.8465)
<20/40:<20/200	0.8093±0.2408 (0.6638–0.9548)
*p* value (Kruskal-Wallis test)	<0.001

Acronyms: EQ-5D-3L, three level version of EuroQol-5D; SD, standard deviation; CI, confidence interval; BEVA, best-eye visual acuity; WEVA, worst-eye visual acuity

## Discussion

In this study, we have shown that sociodemographic factors including age, sex, education, employment status, and household income are important contributors to the quality of life among AMD patients. Furthermore, patients with high BEVA tended to have greater utility values compared to those with low BEVA, regardless of WEVA.

To our knowledge, only one other study investigated utility values for AMD patients within an Asian population [8]. In 2012, Au Eong and colleagues revealed that the mean EQ-5D score for 338 AMD patients was 0.89 [8]. This score is similar to our mean EQ-5D-3L score of 0.8765, which is significantly higher than that from studies within Western population. In a multi-center study investigating EQ-5D scores for AMD patients, Soubrane and colleagues showed that the mean EQ-5D score for 401 patients from France, Germany, Spain, the United Kingdom, and Canada was 0.65 [9]. Similarly, Lotery and colleagues revealed that the mean EQ-5D score for AMD patients from the United Kingdom was 0.67 [10].

Several reasons for the apparent difference in utility values of AMD patients among Western and Asian populations may exist. First, polypoidal choroidal vasculopathy (PCV), an AMD variant, appears to be more common among Asian populations, while neovascular AMD is more common in Western populations [6]. While neovascular AMD leads to progressive scarring, the natural history of PCV is characterized by less scarring, which could lead to less severe visual impairment among Asian AMD patients [6]. Second, the innate differences in sociocultural factors may have contributed to the patients’ perception of the disease, as well as coping strategies, which could in turn result in differing utility scores according to region [11]. Specifically, it has been previously suggested that the fact that elderly adults, the main population susceptible to AMD, have a greater tendency to reside alone or with only one person among Western regions may contribute to the lower utility values within Western populations [12]. As residing alone may lead to lower quality of life among elderly adults, such sociocultural factors may have contributed to the lower EQ-5D scores for AMD patients in Western populations compared to that from our study [8]. Indeed, higher utility scores for Asian populations compared to those for Western populations are also observed among not only AMD patients, but also glaucoma, diabetic retinopathy, and myopia patients [13–16].

Only a few studies have investigated the effect of sociodemographic factors on utility values among AMD patients. In a study investigating the quality of life for diabetic retinopathy and AMD patients, Brown and colleagues have shown that among age, sex, and visual acuity, only visual acuity was associated with quality of life among AMD patients [17]. This is in contrast to the results from our study, in which both age and sex, along with visual acuity, was also associated with utility values. Although the exact reasons for this discrepancy are unknown, evidence from studies of utility values among the general population according to sociodemographic characteristics seem to be more in line with the results from our study. For example, greater age, lower education, unemployment, and lower household income may be associated with decreased psychosocial well-being, an important factor that contributes to the quality of life among AMD patients [18]. In our study, particularly as most patients did not experience vision loss, sociodemographic factors such as education and employment have been shown to be important drivers of the quality of life among AMD patients.

Previous studies are in line with the results from our study depicting decreased utility values for those with worse visual acuity. In a study determining the impact of AMD on quality of life, Espallargues and colleagues showed that visual acuity was significantly associated with utility values among AMD patients [19]. Similarly, Brown and colleagues conducted a study investigating the association between BEVA and utility values among AMD patients and revealed that utility values among AMD patients are highly dependent on the degree of BEVA [20]. Finally, in a study investigating the association between BEVA, WEVA, and utility values among AMD patients, Sahel and colleagues depicted that both BEVA and WEVA influenced quality of life, with BEVA contributing more significantly to general vision-related quality of life compared to WEVA [21]. Although both BEVA and WEVA were associated with utility, the lower utility values for those with low BEVA regardless of WEVA indicates the importance of managing BEVA in order to maintain quality of life for AMD patients. Indeed, while interventions aimed at minimizing visual acuity reduction on the worst-seeing eye are needed, management of best-seeing eye may be imperative in maintaining the quality of life for AMD patients.

Interestingly, there was no difference in utility values according to AMD subtype. Unlike early AMD, late AMD patients generally undergo therapeutic interventions in order to delay the progress of the disease. Although poorly managed late AMD could lead to reduced visual acuity, late AMD patients did not have significantly lower utility values. One possible explanation for this could be related to the high-level of care late AMD patients receive in Korea. Due to the near-universal health care system and relative ease in which patients can receive care from teaching hospitals, it is reasonable to assume that most late AMD patients in Korea have assess to therapeutic management in a timely manner, which could ultimately lead to utility values similar to those of early AMD patients [22]. However, the exact reasons for the lack of discrepancy in quality of life according to AMD subtype are unknown and merit further investigation.

Several limitations must be considered when interpreting the results from our study. First, utility value was measured by EQ-5D only. Although EQ-5D is a widely-used measure of utility, other modes of utility measurement, such as time trade-off and standard game methods are needed to validate the findings from this study. Second, the cross-sectional design of the study only reveals the association between sociodemographic and clinical characteristics with utility values. Therefore, future studies with longitudinal designs are needed. Third, other clinical factors that may affect utility values, such as contrast sensitivity, were not included in our study due to the lack of information. Finally, there may not have been enough participants with vision loss and late AMD to adequately determine the difference in utility values according to AMD subtype and vision loss. For example, power calculation for the number of participants with vision loss required revealed 479 patients (for alpha and power values of 0.05 and 0.80, respectively), which is far larger than the 48 patients with vision loss in our study. Future studies with greater numbers of AMD patients with vision loss and late AMD are required to adequately determine whether differences in utility values exist according to AMD subtype and vision loss.

Despite these limitations, a number of strengths exist. First, the relatively large study population and nationally-representative data enhance the generalizability of our findings. Second, we took into account several factors in addition to visual acuity, including age, sex, household income, education, employment, and martial status, revealing that such sociodemographic factors are important contributors in determining the quality of life for AMD patients. Third, we conducted the study within an Asian population, a study group little-studied previously.

## Conclusions

Sociodemographic factors are important contributors to the quality of life for AMD patients in Korea. Furthermore, high BEVA, regardless of WEVA, was associated with greater utility value. Preventing BEVA reduction may be important in maintaining utility values for AMD patients.
