# Correction: Utility values for age-related macular degeneration patients in Korea

**DOI:** 10.1371/journal.pone.0209519

**Published:** 2018-12-14

**Authors:** Seulggie Choi, Sang Min Park, Donghyun Jee

In the Funding section, the grant number from the funder Ministry of Health & Welfare, Republic of Korea is listed incorrectly. The correct grant number is: HC17C0133.
